# The Factors Affecting the Refusal of Reporting on Medication Errors from the Nurses' Viewpoints: A Case Study in a Hospital in Iran

**DOI:** 10.1155/2013/876563

**Published:** 2013-04-09

**Authors:** Mohammadkarim Bahadori, Ramin Ravangard, Amin Aghili, Jamil Sadeghifar, Mahdi Gharsi Manshadi, Javad Smaeilnejad

**Affiliations:** ^1^Health Management Research Centre, Baqiyatallah University of Medical Sciences, Tehran, Iran; ^2^School of Management and Medical Information Sciences, Shiraz University of Medical Sciences, Shiraz, Iran; ^3^Students' Scientific Research Center, School of Management and Medical Information, Tehran University of Medical Sciences, Tehran, Iran

## Abstract

*Objective*. Medication errors are the most common types of medical errors which considerably endanger the patient safety. This survey aimed to study the factors influencing not reporting on medication errors from the nurses' viewpoints in Abbasi Hospital of Miandoab, Iran. *Methods*. This was a cross-sectional, descriptive and analytical study conducted in 2012 in which all nurses (*n* = 100) working in different inpatient units were studied using a consensus method. Required data were collected using a questionnaire. Collected data were analyzed through some statistical tests including Independent *t*-test, ANOVA, and chi-square. Results. According to the results, the most important reasons for not reporting on medication errors were related to the managerial factors (3.56 ± 0.996), factors related to the process of reporting (3.32 ± 0.797), and fear of the consequences of reporting (3.01 ± 1.039), respectively. Also, there was a significant relationship between employment status and fear of the Consequences of reporting on medication errors (*P* < 0.008). *Conclusion*. This study results showed that managerial factors had the greatest role in the refusal of reporting on medication errors. Therefore, for example, establishing a mechanism to improve quality rather than focus only on finding the culprits and blaming them can result in improving the patient safety.

## 1. Introduction

One of the most fundamental components of health care quality is the patient safety [1]. The patient safety is a priority for every health care system which follows the ensuring and improving of the quality of health care [2] and also is one of the main concerns of all health care systems [3, 4]. The principle of “no harm” in the Hippocratic Oath is a confirmation of this issue [5]. The patient safety is known as to avoid injuries to the patients or occurring unexpected adverse events of health care processes [6].

Adverse events and medical errors are the main factors endangering the patient safety which are the most important problems of all health care systems, and all of these systems try to reduce their resulted injuries [5]. Statistics show that from 2.9% to 16.6% of hospital inpatients suffer from treatment-related adverse events [7, 8]. On the other hand, the rate of medical errors throughout the world is so high that is accounted for one of the five major causes of deaths [9]. 

One of the most common types of medical errors is medication error which because of their high prevalence and potential risks to patients, the rate of medication errors is considered as an indicator to determine the level of the patient safety in hospitals [3, 9, 10]. Medication error has been defined as the preventable misuse of the drugs [11, 12].

In the patient safety management issues, from the 1990s onwards, determining the factors influencing the occurrence of errors as well as the barriers to reports on occurred errors has been focused instead of only detection and prevention of errors [13]. The occurrence of medication errors among nurses is more than that among other health care professionals [14]. The results of studies have shown that among approximately 44000 to 98000 annual deaths due to medical errors, 7000 deaths have been related to the medication errors [9]. Reporting on the medication errors can cause maintaining the safety of patients as well as providing a valuable database of information to prevent medication errors in the future [15].

Several studies have been conducted on determining the barriers to report on medication errors. Tol and colleagues in their study on a group of nurses conducted at a teaching hospital concluded that the managerial factors were the most important barriers to report on medication errors [16]. The results of Wakefield and colleagues' study on 1300 nurses showed that using medication errors for reprimanding nurses by their supervisors was one of the factors affecting the refusal of reporting on medication errors [17]. Based on the results of a study conducted in Japan, the main cause of occurring medication errors among newly graduated nurses was related to the wrong administration of intravenous drugs which, in turn, was due to their little knowledge of pharmacology [18].

The researchers of the present study in their searches found that most of the researches had been focused only on identifying the causes of medication errors and few researches had conducted to determine the factors influencing the refusal of reporting on medication errors or barriers to reporting on these errors. Because determining the factors influencing not reporting on the medication errors can result in taking appropriate managerial and individual measures to increase the rate of reporting on medication errors and finally decrease the occurrence of these errors, the present survey aimed to study the factors influencing not reporting on medication errors from the nurses' viewpoints in Abbasi Hospital of Iran.

## 2. Methods

This was a cross-sectional, descriptive analytical study conducted in 2012 in which all nurses (*n* = 100) working in different inpatient units of Abbasi Hospital in Miandoab, an Iranian hospital affiliated to Urmia University of Medical Sciences, were studied using a consensus method.

Required data were collected using a questionnaire consisting of two sections: the first section included nurses' demographic characteristics such as age, sex, marital status, education level, employment status, types of work shifts, experience, and their service unit. The second section, also, included 19 questions in 3 domains: fear of the consequences of reporting (with 11 items), managerial factors (with 5 items), and factors related to the process of reporting (with 3 items). The five-point Likert scale was used to determine the factors affecting the refusal of reporting on medication errors, whereby 1 refers to strongly disagree and 5 as strongly agree. Though the validity and reliability of this questionnaire had been confirmed in Tol and colleagues' study [16], in the present study, the reliability of this questionnaire was approved again (*α* = 0.82). For collecting data, one of the researchers attended the mentioned hospital in three work shifts—morning, evening, and night—and distributed the questionnaire among studied nurses after obtaining oral consent from them for participating in this study and explaining the objectives of the study and assuring the confidentiality of collected data.

Finally, collected data were analyzed using SPSS 19.0 through some statistical tests including independent *t*-test, ANOVA, and chi-square. *P* < 0.05 was considered statistically significant.

## 3. Results

The response rate was 83%. The results showed that among 83 nurses who participated in this study, most of them (*n* = 65, 78.3%) were female, 71% were married (*n* = 59), 55.5% were in the 25–35 age group (*n* = 46), 86.8% had a bachelor's degree (*n* = 72), 49.4% were employed officially (*n* = 41), 38.6% had lower than 5 years job experience (*n* = 32), 88% were working in rotating work shifts (*n* = 73), and 39.7% were working in the Intensive Care Units (*n* = 33) (Table 1).

The mean self-reported scores of studied domains for not reporting on medication errors were as follows.

Fear of the consequences of reporting (3.01 ± 1.039), managerial factors (3.56 ± 0.99), and factors related to the process of reporting (3.32 ± 0.79). Therefore, managerial factors were the most important reason for not reporting on the medication errors.

The results showed that the highest mean score in the managerial factors domain was related to “the heads' focus only on finding the culprits and blaming them, regardless of other factors involved in the occurrence of errors” (3.674 ± 1.21). The highest mean scores in the domain of fear of the consequences of reporting was related to “fear of judicial affairs following reporting on medication errors” (3.68 ± 1.21), and in the domain of factors related to the process of reporting was associated with “lack of a clear definition of medication errors” (3.144 ± 1.29).

The frequency, mean, and standard deviation of nurses' responses to the questions related to each studied domains (fear of the consequences of reporting, managerial factors, and factors related to the process of reporting) and their items have been shown in Table 2 based on their importance in occurring medication errors from the nurses' viewpoints.

Also, the results showed that there were not any significant relationships between some nurses' demographic characteristics (such as marital status, types of work shifts, age, education level, employment status, and service units) and the studied domains of not reporting on medication errors (including fear of the consequences of reporting, managerial factors, and factors related to the process of reporting) (*P*-value >0.05). However, there was a significant relationship between employment status and fear of the consequences of reporting on medication errors (*P* < 0.008).

## 4. Discussion

Identification and reduction of medication errors requires a system to be designed for finding the root causes of occurring them. Based on such a system, first the occurred errors should be reported, and then, the major causes of occurring them should be found in an intimate and scientific situation. In this regard, the present survey aimed to study the factors influencing not reporting on medication errors from the nurses' viewpoints in Abbasi Hospital, Miandoab.

The study results showed that managerial factor was the most one causing not reporting on medication errors, and other factors including factors related to the process of reporting and fear of the consequences of reporting had the next priorities for not reporting on medication errors from the viewpoint of nurses. Tol and colleagues in a similar study conducted among the nurses of Baharloo hospital in Tehran also found the same results [16]. The similarity of these two studies in using the same methods and population studies (viz., the nurses of public hospitals) is a reason for those similar results. Kouhestani and Baghcheghi in their study on the nursing students reported the fear of the consequences of reporting errors as the most important factors of not reporting on medication errors by them [19]. The different population study of mentioned studies (viz., nursing student and nurses) can be a reason for their different results. In another study carried out by Osborne and colleagues the most important reasons for not reporting on medication errors were fear of being blamed, fear of being labeled as incompetent nurses and inadequacy, fear of their future professional career, fear of judicial issues, and adverse reactions of their heads and colleagues [20].

According to the results of the present study, among the studied managerial variables, “the heads' focus only on finding the culprits and blaming them, regardless of other factors involved in the occurrence of errors,” had the greatest impact on not reporting on medication errors from the viewpoints of nurses. The results of the Tol and colleagues' study [16] as well as Hosseinzadeh and colleagues' study [21] confirm the present study results.

In the current study, unlike other researches in this field [3, 15, 17, 22], the factor of fear of the consequences of reporting had approximately the same mean score as the factor of managerial. Among the studied variables in the factor of fear of the consequences of reporting, “fear of judicial issues following reporting on medication errors” was introduced as the most important variable influencing not reporting on medication errors.

One of the new trends that are emerging today and should be paid particular attention is patients' litigation against possible and unwanted medical errors and malpractices throughout the diagnosis and treatment processes [23]. Kouhestani and Baghcheghi in their study had reported “fear of the impact of reporting of errors on the students' evaluation scores and their educational outcomes” as the most important variable in the factor of fear of the consequences of reporting which does not confirm the present study results. The difference between the population studies of these two studies can be a reason for the results differences [19]. However, the results of Tol and colleagues' study confirm this result of the present study [16].

Among the studied variables related to the process of reporting, “lack of a clear definition of medication errors” was the most important variable influencing not reporting on medication errors from the studied nurses' viewpoints. Hosseinzadeh and colleagues showed similar results in their study [21]; however, the results of Tol and colleagues' study [16] as well as Kouhestani and Baghcheghi's study [19], which had introduced “not paying attention to the reporting on some medication errors” as the most important reason for not reporting on medication errors, do not confirm the present study results in the mentioned domain.

Nurses are the second group protecting against medication errors because they play an important role in the distribution, preparation, and administration of medication orders [24].

Nurses' proper and appropriate function of reporting on medication errors will prevent doing possible harm to the patients and also is considered as a source of valuable information to avoid occurring similar errors in the future and finally can dramatically provide safety for patients [15]. In other words, it can be said that as a rule, making errors is inevitable; however, reporting them and following their subsequent harm can prevent recurrence of those errors for the other patients [25].

The present study had some limitations such as studying only on one hospital and a small sample as well as its limitation of results generalizability. Therefore, it is suggested that similar studies should be carried out on other public and also private hospitals using large samples and population studies, and then those results should be compared with the present study results. Also, future studies should be conducted using interventional designs to identify the major causes of occurring medication errors, other reasons for not reporting them, and strategies to prevent or reduce their occurrence.

## 5. Conclusion

The current study results showed that managerial factors had the greatest role in the refusal of reporting on medication errors. Therefore, designing a system for reporting on medication errors properly and accurately, training nurses in the quality of reporting on medication errors, and above all, establishing a mechanism to improve quality rather than focus only on finding the culprits and blaming them can result in more reporting on medication errors, reducing their occurrence, and, finally, improving patient safety.

## Figures and Tables

**Table 1:** The demographic characteristics of studied nurses.

Variables	Frequency (%)
Age (year)	
Less than 25	10 (12%)
25–35	46 (55.5%)
35–45	15 (18.1%)
More than 45	6 (7.2%)
Missing	6 (7.2%)
Sex	
Male	17 (20.5%)
Female	65 (78.3%)
Missing	1 (1.2%)
Marital status	
Single	23 (27.8%)
Married	59 (71%)
Missing	1 (1.2%)
Education level	
Diploma	7 (8.4%)
Associate degree	4 (4.8%)
Bachelor's degree	72 (86.8%)
Employment status	
Official employee	41 (49.4%)
Contractual employee	29 (34.9%)
Other	12 (14.5%)
Missing	1 (1.2%)
Types of work shift	
Fixed shifts	10 (12%)
Rotating shifts	73 (88%)
Job experience (year)	
Less than 5	32 (38.6%)
5–10	27 (32.5%)
More than 10	17 (20.5%)
Missing	7 (8.4%)
Service unit	
General Care Units	23 (27.8 %)
Intensive Care Units	33 (39.7%)
Missing	27 (32.5%)

**Table 2:** The frequency (%), mean, and standard deviation of nurses' responses to the variables based on their importance in occurring medication errors from their viewpoints.

Factors	Variables	Scale					Mean ± SD *F* (%)	Mean ± SD total
		Strongly agree *F *(%)	Agree *F* (%)	neutral *F* (%)	Disagree *F* (%)	Strongly disagree *F* (%)		
Fear of the consequences of reporting	Fear of the impact of reporting of errors on the personnel's annual evaluation	13 (15.9%)	19 (23.2%)	8 (9.8%)	30 (36.6%)	12 (14.6%)	3.11 ± 1.35	3.01 ± 1.039
	Fear of the impact of reporting of errors on their salary and benefits	10 (12.2%)	25 (30.5%)	11 (13.4%)	28 (34.1%)	8 (9.8%)	2.99 ± 1.24	
	Fear of being blamed by nursing heads	6 (7.2%)	16 (19.3%)	7 (8.4%)	34 (41%)	20 (24.1%)	3.55 ± 1.25	
	Fear of being blamed by doctors	9 (11%)	18 (22%)	7 (8.5%)	33 (40.2%)	15 (18.3%)	3.33 ± 1.30	
	Fear of being blamed by colleagues	16 (9.8%)	31 (38.3%)	13 (16%)	16 (19.8%)	5 (6.2%)	3.54 ± 1.19	
	Fear of producing side effects in patients	11 (13.8%)	14 (17.5%)	2 (2.5%)	29 (36.3%)	24 (30%)	3.51 ± 1.43	
	Fear of being labeled as incompetent nurses and inadequacy	14 (17.1%)	17 (20.7%)	8 (8.9%)	32 (39%)	11 (13.4%)	3.11 ± 1.35	
	Fear of colleagues' behavior	13 (15.9%)	27 (32.9%)	14 (17.1%)	19 (23.2%)	9 (11%)	2.8 ± 1.27	
	Fear of expressing a negative attitude towards the nurse(s) making errors by the patient and his/her family	5 (6.2%)	17 (21%)	7 (8.6%)	39 (48.1%)	13 (16%)	3.47 ± 1.17	
	Fear of judicial issues following reporting on medication errors	6 (7.4%)	11 (13.6%)	7 (8.6%)	36 (44.4%)	21 (25.9%)	3.68 ± 1.21	
	Fear of informing colleagues working in other units and other facilities about one's medication error	4 (4.9%)	19 (23.5%)	8 (9.9%)	34 (42%)	16 (19.8%)	3.48 ± 1.19	

Managerial factors	Lack of receiving positive feedback from the nursing heads following to report on medication errors	4 (4.8%)	17 (20.5%)	10 (12%)	28 (33.7%)	24 (28.9%)	3.61 ± 1.24	3.56 ± 0.99
	False beliefs in nursing heads and managers	5 (6%)	19 (22.9%)	12 (14.5%)	27 (32.5%)	20 (24.1%)	3.46 ± 1.25	
	The heads' focus only on finding the culprits and blaming them, regardless of other factors involved in the occurrence of errors	5 (6%)	12 (14.5%)	12 (14.5%)	30 (36.1%)	24 (28.9%)	3.67 ± 1.21	
	Disproportionate reactions of the heads to the error seriousness	4 (4.8%)	20 (24.1%)	2 (2.4%)	35 (42.2%)	22 (26.5%)	3.24 ± 1.61	
	Disproportionate reactions of the heads to the error importance	3 (3.7%)	22 (27.2%)	9 (11.1)	25 (30.9)	22 (27.2%)	3.50 ± 1.25	

Factors related to the process of reporting	Not paying attention to the reporting on some medication errors	21 (25.6)	14 (17.1%)	11 (13.4%)	28 (34.1%)	8 (9.8%)	2.85 ± 1.39	3.32 ± 0.79
	Lack of a clear definition of medication errors	8 (9.6%)	25 (30.1%)	11 (13.3%)	25 (30.1%)	14 (16.9%)	3.14 ± 1.28	
	To forget reporting on the medication errors	13 (15.7%)	20 (24.1%)	11 (13.3%)	28 (33.7%)	11 (13.3%)	3.05 ± 1.32	
